# Connecting the Dots of a Rare Connective Tissue Disease: Pseudoxanthoma Elasticum

**DOI:** 10.7759/cureus.18481

**Published:** 2021-10-04

**Authors:** Ashley L Yenior, George Pujalte, Jeff Nadwodny, Lorena C Costa, Richard J Presutti

**Affiliations:** 1 Department of Family Medicine, Mayo Clinic, Jacksonville, USA; 2 College of Medicine, Santa Casa de Misericordia de Vitoria, Vitoria, BRA

**Keywords:** family history, genetic counseling, intervention, multidisciplinary, connective tissue disorder, autosomal recessive

## Abstract

Pseudoxanthoma elasticum (PXE) is a rare, autosomal recessive connective tissue disease that manifests primarily in the skin, eyes, vasculature, and gastrointestinal tract. Most cases occur in women and are present in the third decade of life. Diagnosis is confirmed via skin biopsy or by genetic testing that reveals a variant *ABCC6* gene. We present the case of a 68-year-old woman who came to the clinic to discuss her daughter’s diagnosis of PXE, specifically, what testing she and her family should pursue. A family pedigree revealed a strong family history of abdominal aortic aneurysm (AAA). Although PXE has not been directly related to AAA, this raised concern for familial connective tissue disease. It was recommended that all family members undergo AAA screening with ultrasound, but that not all family members warranted genetic testing. Patients diagnosed with PXE should establish care with specialists to monitor for adverse outcomes.

## Introduction

Pseudoxanthoma elasticum (PXE) is a rare connective tissue disease characterized by dystrophic mineralization of elastic fibers that can affect primarily the skin, eyes, cardiovascular, and gastrointestinal tract [1,2]. It is an autosomal recessive condition with a molecular mutation in the *ABCC6* gene, located on the short arm of chromosome 16 [3]. It demonstrates incomplete penetrance where the estimated incidence is approximately one in 25,000 to 100,000 people, with women accounting for two-thirds of cases [2,4,5]. PXE typically presents as xanthomatous skin lesions, which should indicate a skin biopsy with genetic testing, if needed [1,2].

In this case, a 68-year-old woman discusses her daughter’s diagnosis of PXE, specifically, whether she and her family should pursue genetic testing. A family pedigree showed a history of abdominal aortic aneurysm (AAA) in both the patient’s and her husband’s families. Although PXE has not been directly related to AAA [6], this raised concern for a familial connective tissue disease due to the presence of two well-known heritable disorders that affect the connective tissue in the same family. A multidisciplinary approach and annual monitoring of her relatives were recommended.

## Case presentation

**Figure 1:**
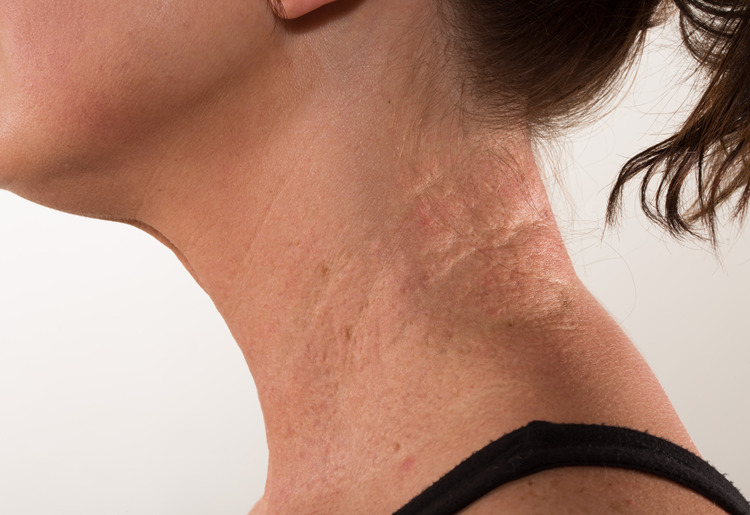
Representative image of xanthomatous skin lesion, a common presentation of PXE

**Figure 2:**
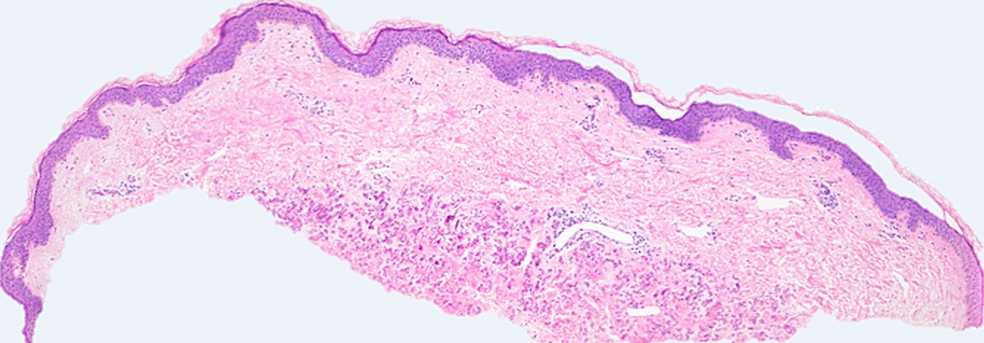
Histopathological image of the patient's daughter's xanthomatous lesion, consistent with pseudoxanthoma elasticum

**Figure 3:**
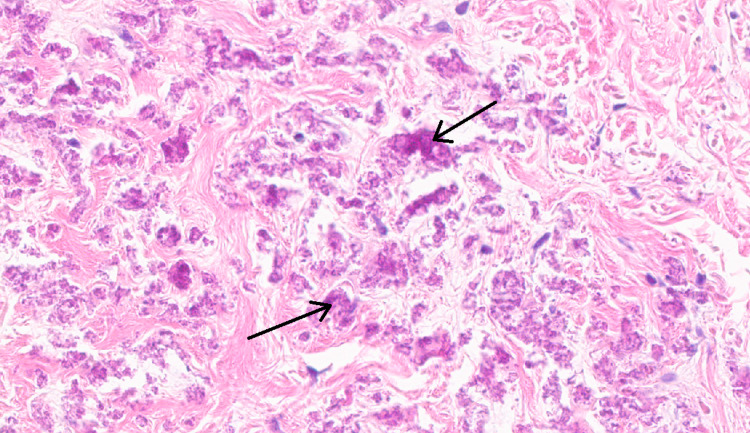
A magnified view of the histopathological image in Figure 2, with arrows identifying the fragmented elastic fibers of a pseudoxanthoma elasticum lesion

A 68-year-old woman presented to discuss whether genetic testing for her family would be appropriate, since her daughter (age 35) had been recently diagnosed with PXE. The patient's daughter presented to a plastic surgeon with a scalp lesion. Subsequently, a lesion similar to that in Figure 1 was identified on her lateral neck. The lesion was biopsied, and the histopathological results (Figures 2, 3) were consistent with PXE. As seen in Figure 3, the soft tissue becomes mineralized leading to fragmentation of elastic fibers [7]. The mechanism of this change is unknown [7].

**Figure 4:**
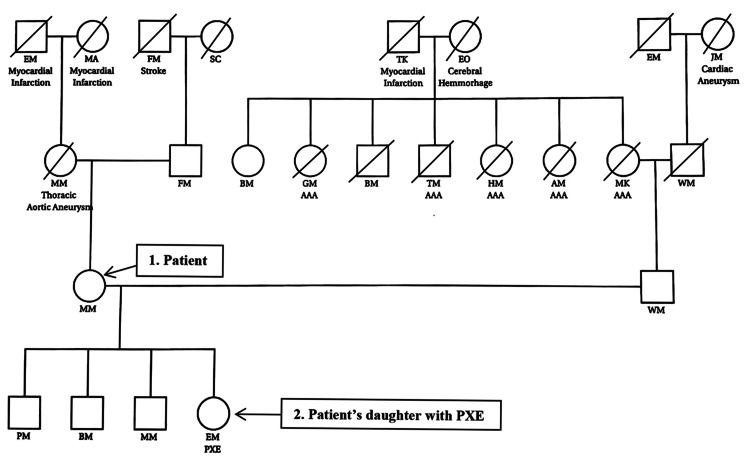
Family pedigree AAA: Abdominal Aortic Aneurysm, PXE: Pseudoxanthoma Elasticum

After careful family history inquiry, a family pedigree (Figure 4) showed a strong history of aneurysms in the patient’s and her husband’s families. There was no known history of parental consanguinity or Jewish ancestry. She inquired about the next steps for herself, her husband, and their children.

The patient did not have xanthomatous skin findings and denied having angina, claudication, or changes in vision. Since the majority of PXE-related symptoms present in the third decade of life [2], a decision was reached that genetic testing was unnecessary for herself and her husband. The patient's daughter, who has confirmed PXE, established care with an internist, cardiologist, and ophthalmologist. Her other children considered genetic testing for PXE and established care with appropriate specialists to be evaluated for the disease.

It was recommended that all family members undergo AAA screening with ultrasound due to their strong family history, and an echocardiogram was advised to evaluate for an aneurysm. Cardiovascular screening for the patient did not find signs of an aneurysm.

The most common adverse outcome of PXE is macular scarring and hemorrhage that may precede cutaneous signs [2]. This suggests that family members of diagnosed patients should undergo ophthalmologic screening as well. Ophthalmologic screening for the patient was negative.

## Discussion

PXE is a multisystem connective tissue disorder that affects elastic fibers, presenting characteristically with skin lesions (typically yellowish papules) generally located on the lateral neck (Figure 1) and flexural surfaces [4], and with ocular involvement (macular scarring, hemorrhage, and angioid streaks of the retina) [1,2]. PXE can also affect multiple organ systems including the cerebrovascular system leading to stroke and transient ischemic attacks [1], the cardiovascular system leading to claudication, angina, and myocardial infarction [2], the renovascular system leading to hypertension [2], and the gastrointestinal system leading to upper gastrointestinal bleeding and mesenteric ischemia [1,2,8].

Dermatologic changes are typically the first presenting symptom and can appear as early as five years old, whereas retinal changes develop later in life but are typically seen on routine eye examinations as early as 10-30 years old [2]. Diagnosis of PXE is therefore challenging as the initial diagnostic cutaneous findings are subtle and diagnosis usually occurs later in life when ocular and vascular complications occur [2,9].

Diagnostic criteria of PXE were first proposed at a Consensus Conference in 1992 and have been updated numerous times, including most recently in 2014 [9]. Definitive PXE can be diagnosed with two pathologic mutations of the *ABCC6* gene or ocular findings in an individual less than 20 years old such as angioid streak or peau d’orange [9]. This criterion must be seen together with characteristic PXE skin changes including papules and plaques on the neck or flexural creases and histologic evidence on skin biopsy including calcified elastic fibers in the dermis with positive calcium stain [9].

An AAA is also a connective tissue disorder associated with the degradation of elastin and collagen fibers. Biochemical and environmental factors are known to be relevant and familial predisposition is well recognized. AAA may occur in isolation or as part of a heritable syndrome [10]. According to the family pedigree, familial AAA occurs in the patient's husband's family, on account of more than one family member is affected. As for the patient’s family, it is not possible to confirm if the aneurysm occurrence is heritable or sporadic since only her mother was affected. The relationship between PXE and AAA has not yet been established but is well known that both conditions are heritable connective tissue disorders that can involve alteration in the elastic fibers of the vasculature [2,10].

The diagnosis and management of PXE require a multidisciplinary approach. Recommendations based on expert opinion outline that at the time of diagnosis, patients should undergo a complete skin check, a dilated eye examination, a baseline echocardiogram, a stress test, and a Doppler ultrasound of the peripheral vasculature. Annual monitoring should include an ophthalmic and cardiovascular examination [2].

Due to the genetic inheritance of PXE, it is appropriate to offer genetic counseling for those who are affected, are carriers, or are at risk of being carriers with prenatal or preimplantation genetic testing for pregnancies at increased risk [2]. Pregnant patients with PXE will typically have normal pregnancies as it is not associated with a markedly increase in fetal loss and there is a low incidence of gastrointestinal bleeding and retinal complications [2].

Primary Care Physicians can help manage patients with PXE including ensuring they have appropriate annual screening completed and follow up with specialists and provide lifestyle counseling regarding weight control and smoking cessation to lower their risk of cardiovascular disease. Patients should be counseled to avoid contact sports due to the risk of retinal hemorrhage with head trauma [2]. Medications that may precipitate gastrointestinal bleeding should be avoided [2]. Some studies have investigated the use of magnesium, bisphosphonates, and phosphate binders as treatment of PXE; however, this research has been limited and many have not been shown to have statistical significance. Further larger scale and long-term studies are needed in this potential treatment [11].

## Conclusions

PXE is a rare autosomal recessive connective tissue disorder, generally presenting in the third decade of life, that manifests primarily through skin lesions, angioid streaks of the retina, cardiovascular disease (e.g., aneurysms), and gastrointestinal bleeding. Diagnosis can be confirmed by dermatopathology or genetic testing. Affected patients should establish care with specialists to monitor for the aforementioned adverse outcomes. If appropriate precautions are taken and routine monitoring is performed, patients with PXE can live normal lives.

The occurrence of PXE and familial AAA in the same family should raise concern for the presence of familial connective tissue disease. Meanwhile, continued progress in understanding the pathophysiology of PXE may provide novel strategies to counteract this currently intractable condition and to better understand the relationship with other connective tissue disorders. Finally, it is important for Primary Care Physicians to understand the possible evaluation and testing needed for family members if a patient's close relative were to be diagnosed with PXE.
